# Role of DZ2002 in reducing corneal graft rejection in rats by influencing Th17 activation via inhibition of the PI3K/AKT pathway and downregulation of TRAF1

**DOI:** 10.1515/biol-2025-1214

**Published:** 2026-01-01

**Authors:** Qian Cao, Yong Li, Yongqian Zhang, Shuang Tan, Junjun Long, Ermiao Tian, Jie Dong, Lan Li

**Affiliations:** Department of Ophthalmology, The Affiliated Calmette Hospital of Kunming Medical University, Kunming 650000, China

**Keywords:** corneal transplantation, DZ2002, homocysteine, PI3K/AKT signaling pathway, rejection, TRAF1

## Abstract

To investigate the mechanism of DZ2002, a reversible type III S-adenosyl-l-homocysteine hydrolase (SAHH) inhibitor, in alleviating corneal graft rejection in rats through the downregulation of TRAF1 and modulation of Th17 differentiation, so as to provide a new therapeutic strategy for corneal graft rejection. Rat models of corneal graft rejection were constructed and treated with DZ2002 eye drops. Metabolomics and transcriptomics sequencing analyses were used to identify the key metabolite, homocysteine, and its associated gene, TRAF1. Histological assessments were conducted to evaluate the effect of DZ2002 on corneal graft rejection. Additionally, the underlying mechanism of action was explored through Gene Ontology (GO) and the Kyoto Encyclopedia of Genes and Genomes (KEGG) analyses. Homocysteine emerged as a key metabolite involved in corneal graft rejection, and DZ2002 could significantly decrease the rejection index and reduce the opacity and edema of corneal grafts. The effect of DZ2002 in alleviating rejection was attributed to the downregulation of TRAF1 expression, inhibition of the PI3K/AKT pathway, decreased expression of chemokine CCL5 in the aqueous humor, and suppression of the differentiation of T cells into Th17 cells. DZ2002 was effective in reducing corneal graft rejection in rats by downregulating TRAF1 and modulating Th17 differentiation.

## 1 Introduction

Corneal blindness is a type of ocular disease that leads to corneal dysfunction and loss of vision stemming from a variety of etiologies, including keratitis, corneal degeneration, and corneal chemical injury [1]. Recent statistics indicate a substantial global burden, with about 217 million people suffering from moderate to severe vision impairment and an estimated 36 million individuals affected by blindness [2]. Notably, China ranks among the countries with the highest prevalence of blindness, with an estimated six to nine million people with bilateral blindness and about four million patients with corneal blindness who are in urgent need of surgery, with the incidence of corneal blindness escalating at a rate of 10 % per year [3].

Corneal transplantation remains the primary recourse for patients with corneal blindness in an attempt to restore vision and achieve better outcomes [4], 5]. However, despite the presence of anterior chamber immune deviations in the cornea, the 10–50 % incidence of allogeneic corneal transplantation rejection can lead to poor vision correction or even a relapse of blindness [6]. Corneal graft rejection is a complex immune response involving multiple factors, and its mechanism remains poorly understood. At present, glucocorticoids and immunosuppressants used in clinical practice can improve corneal graft rejection, but fail to completely and effectively control rejection or avoid side effects including hypertension, osteoporosis, and glaucoma blindness caused by long-term medication regimens. Therefore, exploring the mechanisms of corneal graft rejection and identifying possible treatment measures to prevent rejection assumes significance.

Protein metabolism during rejection is considerably different from that observed in a normal cornea with long-term inflammation resulting in fibrosis of the corneal tissue, thereby affecting corneal transparency. Metabolomics is an emerging field in the study of corneal diseases that provides new perspectives and analytical tools for uncovering the pathogenesis, diagnosis, and treatment of corneal diseases. The aim of the current study was to explore the mechanism of the metabolite homocysteine (Hcy) in the form of DZ2002, a reversible S-adenosyl-l-homocysteine hydrolase (SAHH) inhibitor, in corneal graft rejection. This can generate insights related to the modulation of corneal metabolism and facilitate the development of new therapeutic drugs for corneal graft rejection.

## 2 Materials and methods

### 2.1 Experimental animals

SPF Wistar female rats (weighing 180–200 g) and SD female rats (weighing 180–200 g) were purchased from Hunan SJA Laboratory Animal Co. (Certificate No. 43004700043639, China), license number SCXK (Beijing) 2019-0010. The rats were randomly divided into two groups: the blank control group (which received no treatment) and the corneal graft rejection group (where a graft rejection model of allogeneic penetrating keratoplasty was established). The right eyes were chosen for surgery, while the left eyes remained untreated so that the postoperative feeding of the rats would not be affected. The use and feeding of the experimental animals were in accordance with the *Regulations for the Administration of Affairs Concerning Experimental Animals* approved by the State Science and Technology Commission.

**Ethical approval:** The research related to animal use has been complied with all the relevant national regulations and institutional policies for the care and use of animals, and has been approved by the Ethics Committee of Affiliated Calmette Hospital of Kunming Medical University (No.YLS2023-67).

### 2.2 Establishment of the penetrating keratoplasty graft rejection model

Initially, tropicamide eye drops were administered to both the donor (Wistar female rats) and recipient (SD female rats) eyes three times, with each application lasting 5 min. An overdose of anesthesia was then induced via an intraperitoneal injection of 0.5 ml of equal amounts of ketamine and diazepam. Subsequently, corneal grafts were procured from both eyes of Wistar rats (*φ* = 4.25 mm) and preserved in Optisol liquid. SD female rats served as recipients and were administered oxybuprocaine hydrochloride eye drops once.

The recipient cornea was incised at the center with a trephine (*φ* = 4 mm), reaching a depth of 1/2 of the corneal thickness. A 15° puncture knife was used to create a wedge in the anterior chamber, and an appropriate amount of Healon was injected to protect the corneal endothelium and maintain the anterior chamber. The corneal sheet was dissected with VANNAS scissors along the incision, and the donor corneal graft was positioned in the implant bed. Eight intermittent stitches with 10-0 micro-sutures were made, leaving the suture knot exposed. The anterior chamber was rinsed with 0.9 % normal saline to replace the Healon. The needle was flushed, and sterile air was injected to maintain the anterior chamber. The eyelids were sutured with 6-0 sutures after applying tobramycin eye ointment to the conjunctival sac.

Post-surgery, tobramycin ophthalmic ointment was applied once a day to prevent infection. Daily observations of the graft were conducted using a stereomicroscope from the first postoperative day, and three aspects, namely, graft opacity, edema, and angiogenesis, were assessed [7]. Scores of these three parameters were summed up to obtain the rejection index (RI) each day, with rejection defined as RI ≥ 6. The observation endpoint was four weeks post-surgery, after which the rats were euthanized.

Grouping:

- Control group (subconjunctival injection of physiological saline):Control
- Corneal transplant rejection model group (subconjunctival injection of physiological saline):Allo
- Homocysteine inhibitor group (DZ20024 times/day after transplantation in rats):Allo + DZ2002
- TRAF1 knockout group (transplantation of rat subconjunctival injection of TRAF1 knockout adenovirus)
- Homocysteine inhibitor + TRAF1 overexpression group (after transplantation in rats, point DZ2002 and subconjunctival injection of TRAF1 overexpressing adenovirus):Allo + DZ2002 + oe-TRAF1

### 2.3 Hematoxylin and eosin (HE) and terminal deoxynucleotidyl transferase-mediated dUTP nick-end labeling (TUNEL) staining

Tissue samples were collected and immersed in 4 % paraformaldehyde overnight at 4 °C. Subsequently, they were paraffin-embedded after ethanol gradient dehydration (two days each with 70 % ethanol, 96 % ethanol, and 100 % ethanol, respectively). Sections with a thickness of 5 μm were then obtained for HE and TUNEL staining.

### 2.4 Metabolomics sequencing for identifying the key metabolites involved in corneal graft rejection

#### 2.4.1 Sample pretreatment and metabolite extraction

Serum samples, including those for sample preparation quality control (QC), from the corneal graft rejection group and the normal group, with six replicates per group, were analyzed using ultra-performance liquid chromatography-mass spectroscopy (UPLC-MS) after appropriate sample treatment. Metabolites were isolated and detected using the Waters UPLC I-Class Plus (Waters, USA) along with a tandem Q Exactive High Resolution Mass Spectrometer (Thermo Fisher Scientific, USA).

#### 2.4.2 Data preprocessing and quality control (QC)

The results obtained from Compound Discoverer were imported into MetaX for data preprocessing. This preprocessing entailed normalization of the data using the Probabilistic Quotient Normalization (PQN) method to obtain the relative peak area and correction of batch effects utilizing the quality control-based robust LOESS signal correction (QC-RLSC). Compounds with a coefficient of variation (CV) of relative peak area >30 % were eliminated from all QC samples.

#### 2.4.3 Screening of differential metabolites

The screening of differential metabolites involved univariate and multivariate analyses to identify variations between the two biological groups. The overall differences between the two groups were assessed using principal component analysis (PCA) and partial least squares discriminant analysis (PLSDA). Subsequently, the variable important for the projection (VIP) values of metabolites were analyzed through orthogonal partial least squares discriminant analysis (OPLSDA), and the VIP value of PLSDA was adopted if OPLSDA was prone to overfitting. Differential metabolites were screened using fold change and *P* values in univariate analysis, and volcano diagrams were plotted. In this study, differential metabolites were identified as per the following conditions: 1) VIP ≥ 1 in the OPLS-DA model; 2) fold change ≥1.2 or ≤0.83; and 3) *P* value < 0.05.

### 2.5 Surface plasmon resonance (SPR) assay

#### 2.5.1 TRAF1 immobilization

The running buffer was PBST.

The carboxylated surfaces of flow cells Fc2 and Fc1 were activated with 0.4 M EDC and 0.1 M NHS at an injection volume of 170 μl and flow rate of 20 μl/min. TRAF1 protein was then coupled to Fc2 using 100 μg/ml protein in sodium acetate buffer (pH 4.5), with an injection volume of 170 μl and flow rate of 20 μl/min. Finally, both Fc2 and Fc1 were blocked with 1 M ethanolamine, using an injection volume of 170 μl and flow rate of 20 μl/min.

#### 2.5.2 TRAF1–L-homocysteine interaction

The running buffer was PBST. The analyte was dissolved in PBST, and the interaction was analyzed using the Fc2–Fc1 double-referenced channel.

A series of L-homocysteine concentrations (0, 3.9, 7.8, 15.6, 31.2, 62.5, 125, 250, and 500 μM) were injected sequentially, and signal responses were recorded in real time.

### 2.6 Transcriptomics sequencing for identifying the key genes associated with Hcy-induced corneal graft rejection

#### 2.6.1 Experimental procedure

RNA was isolated and purified using TRIzol (Thermo Fisher, 15596018) and tested for integrity using the Bioanalyzer 2100 (Agilent, CA, USA). This was followed by paired-end sequencing performed with the Illumina Novaseq™ 6000 (LC-Bio Technologies Co., Ltd., Hangzhou, China) in PE150 mode.

#### 2.6.2 Data quality control

The raw sequencing data were preprocessed and filtered. Genes were screened based on both fold change (FC) and significance level. Specifically, an FC value ≥ 2 or FC ≤ 0.5 (i.e., the absolute value of log2FC ≥ 1) and the *q* value < 0.05 (|log2fc| ≥ 1 and q < 0.05) were used as the threshold criteria. In cases where multiple groups were compared without FC, genes with a *q* value < 0.05 were considered genes showing statistically significant differences between multiple groups. Subsequently, clean data were obtained after filtering. These data were compared with the reference genome to obtain comprehensive alignment information.

Additionally, using the gene location information specified in the genome annotation file (gtf), the read data between the sequencing data and reference genome as well as the regional distribution data between the sequencing data and reference genomes were compared. (Genome: ftp://ftp.ensembl.org/pub/release-101/fasta/mus_musculus/dna/).

### 2.7 RT-PCR for validating the mRNA expression of differentially expressed genes

Total RNA was extracted manually, ensuring a purity of 1.9–2.0. cDNA was synthesized using reverse transcription and stored in a 4 °C freezer for subsequent use. Quantitative assessment of the RNA of samples was conducted, and the relative expression of mRNA was determined based on the cycle threshold (Ct) values.

### 2.8 Enzyme-linked immunosorbent assay (ELISA) for detecting the expression of interleukin-1β (IL-1β), interleukin-6 (IL-6), and transforming growth factor-β (TGF-β)

The procedure outlined in the enzyme-linked immunosorbent assay (ELISA) kit was meticulously followed for detecting the expression of interleukin-1β (IL-1β), interleukin-6 (IL-6), and transforming growth factor-β (TGF-β), and the absorbance was measured at 450 nm using a microplate reader (BioTek, Elx800).

### 2.9 Western blotting (WB) for detecting protein expression levels

Samples were lysed on ice for 10 min and centrifuged at 14,000 g at 4 °C for 15 min. Protein concentration was measured with the BCA Protein Quantification Kit. A total of 80 μl of protein and 20 μl of 5× protein loading buffer were mixed well and boiled in a water bath for 5 min. SDS-PAGE electrophoresis was then performed. Upon completion of electrophoresis, the proteins were transferred onto a PVDF membrane. The membrane strips were submerged in 1 × TBST and subsequently placed in a blocking solution containing 5 % skimmed milk powder on a shaker at room temperature for 40 min to block non-specific binding sites. Primary antibodies targeting specific proteins (TRAF1, AKT, P-AKT, PI3K, and P-PI3K) were incubated at 4 °C overnight. Secondary antibodies were added and allowed to incubate for 40 min. The membrane strips were then developed and photographed to visualize the protein bands.

### 2.10 Immunohistochemistry (IHC) staining detecting the vascular endothelial growth factor (VEGF) positive rate

Tissue specimens were initially fixed overnight in 4 % paraformaldehyde, paraffin-embedded after ethanol gradient dehydration, and stained for immunohistochemistry in 5 μm-thick sections. Primary antibodies used for immunohistochemistry included rat Vascular Endothelial Growth Factor (VEGF) antibody (Affinity, AF5131) at a dilution of 1:50.

### 2.11 Flow cytometry analysis of the ratio of regulatory T (Treg) and T helper 17 (Th17) cells grouping

- CD4 + T cell group: blank control group.
- HCEC-OE-NC + CD4 + T cell group: Co culture of overexpressing control human corneal epithelial cells (HCEC) and CD4 + T cells.
- HCEC-OE-TRAF1 + CD4 + T cell group: Co culture of HCEC cells overexpressing TRAF1 and CD4 + T cells.
- HCEC-OE-NC + DZ2002 + CD4 + T cell group: Co culture with CD4 + T cells after adding inhibitor DZ2002 to overexpressing control HCEC cells.
- HCEC-OE-TRAF1 + DZ2002 + CD4 + T cell group: HCEC cells overexpressing TRAF1 were co cultured with CD4 + T cells after adding inhibitor DZ2002.

Peripheral blood samples were collected from healthy volunteers to obtain peripheral blood mononuclear cells (PBMCs) by centrifugation. The PBMCs were washed once with phosphate buffer solution (PBS) and incubated for 30 min. The cells were then incubated with 2 μL of Brefeldin A (5 mg/ml) for 4 h to facilitate stimulation. Then cells were washed once with PBS and incubated with FITC-CD at 4 °C for 15 min in the dark. After 30 min of incubation with a permeabilization agent, the cells were treated with PE-IL-17, Perep/Cy5.5-IL4, and APC-IFN-γ monoclonal antibodies at 4 °C for 15 min. Finally, the cells were washed once with PBS, resuspended, and subjected to flow cytometry analysis to determine the proportion of Regulatory T (Treg) and T helper 17 (Th17) cells. Stained samples were acquired on a BD FACSCalibur flow cytometer, collecting 10,000 events per sample, and the results were analyzed using FlowJo software to quantify cellular expression.

### 2.12 Statistical analysis

Prism graphpad was used for statistical analysis. Experimental data were represented as the mean ± standard error (mean ± SEM). The *t*-test was used for comparison of measurement data between groups, and one-way analysis of variance (ANOVA) was used for comparison of measurement data between multiple groups. A *P* value of <0.05 was considered to indicate a statistically significant difference; a *P* value of <0.01 was considered to indicate high statistical significance; and a *P* value of <0.001 was considered to indicate a very significant statistical difference.

## 3 Results

### 3.1 Metabolomics sequencing revealed that Hcy is a key metabolite involved in corneal graft rejection

**Figure 1:**
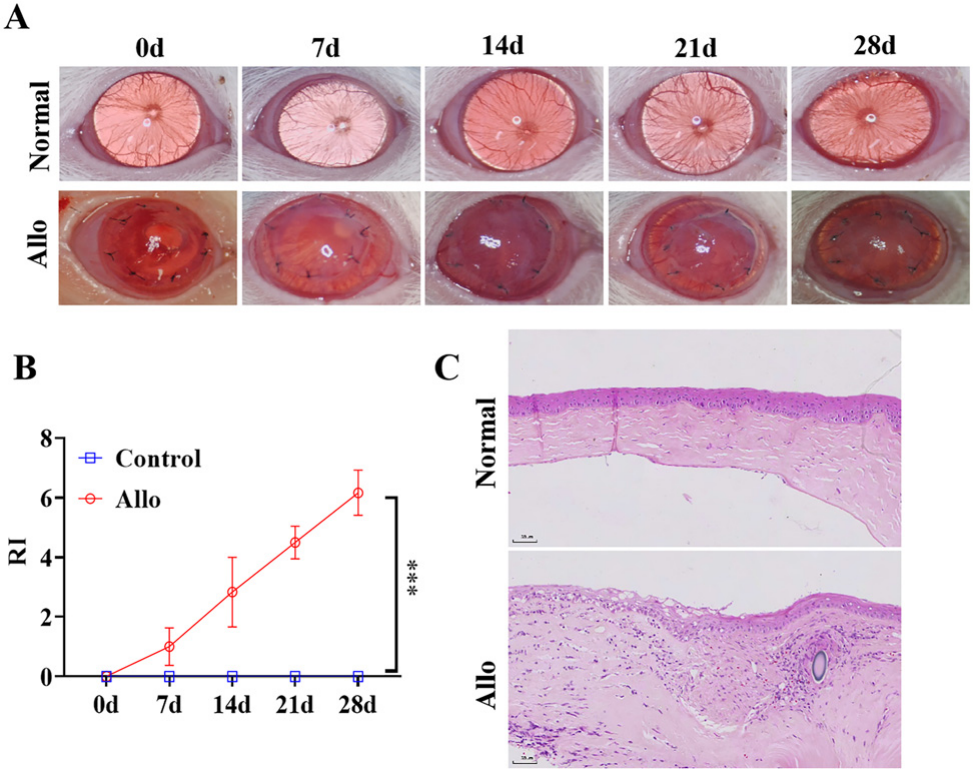
Establishment of the rat corneal transplantation rejection model. (A) Observation of corneal graft rejection under a stereomicroscope. (B) Statistical scoring of rejection severity. (C) H & E staining showing pathological changes in corneal tissue at 4 weeks post-transplantation (200 × magnification).

To study the underlying mechanisms of rejection following a corneal transplantation, we initially established a rat corneal graft rejection model. The corneal graft was observed under a stereomicroscope. After four weeks of transplantation, the graft opacity, stromal edema, and RI scores were significantly increased (Figure 1A and B). HE staining showed that, compared with the cornea of the normal group, the grafts of the rejection group were thicker due to edema, with noticeable infiltration of inflammation cells, indicative of a more pronounced inflammatory response accompanying the rejection of the surgical eye (Figure 1C).

**Figure 2:**
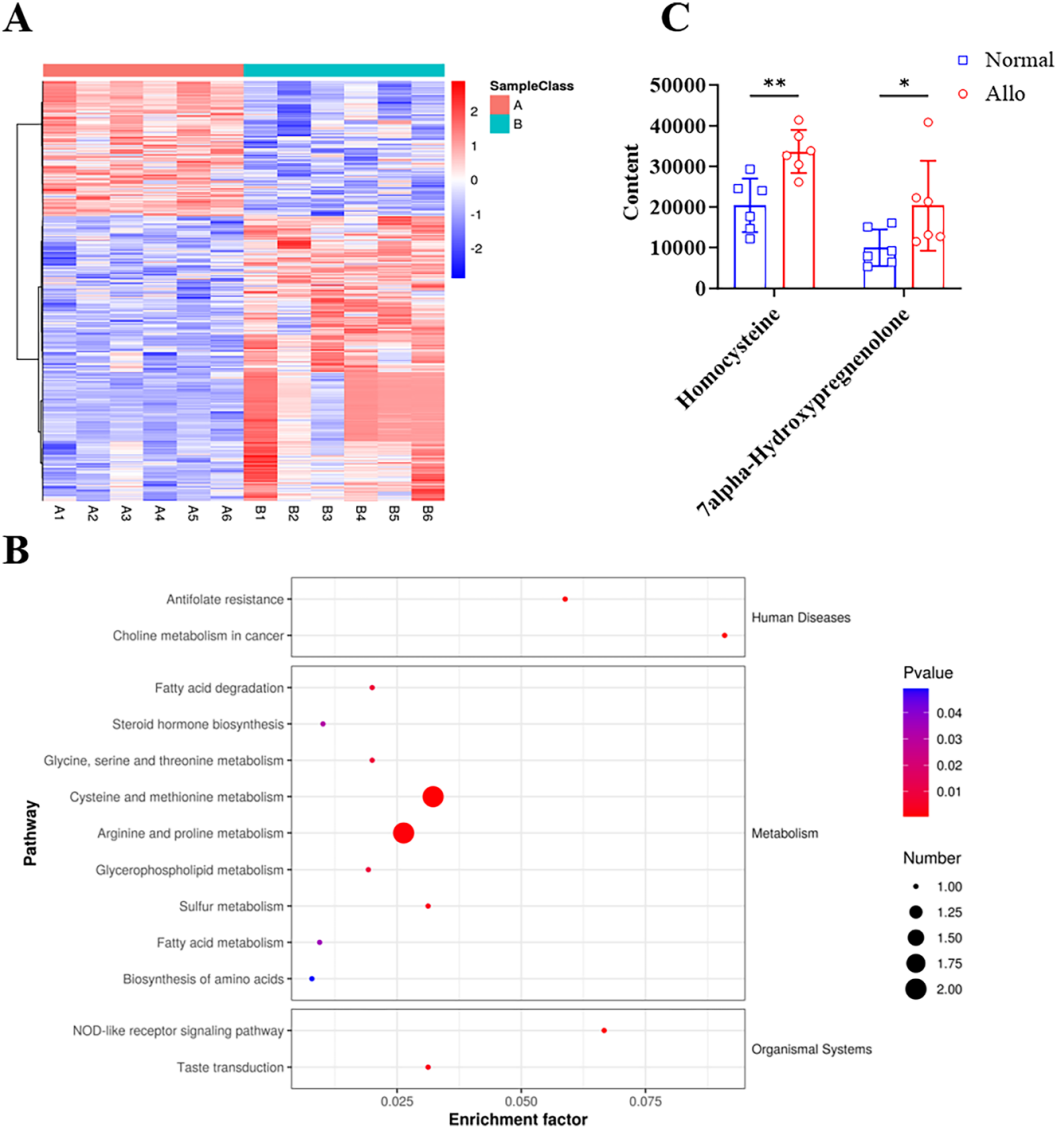
Metabolomic sequencing reveals key metabolites involved in corneal transplant rejection (A) Clustering heatmap of differential metabolites. (B) KEGG enrichment scatter plot of differential metabolites. (C) Content levels of differential metabolites in the corneal transplant rejection group versus the normal control group.

Subsequently, metabolomics sequencing was conducted on serum samples from the rejection group and normal group (with six replicates in each). A total of 40 differential metabolites were screened in the rejection group, of which 25 were found to be upregulated and 15 were downregulated (Figure 2A). KEGG pathway enrichment analysis on the differential metabolites revealed that 240 signaling pathways were enriched, 14 of which were significantly different (*P* ≤ 0.05) (Figure 2B). Notably, two metabolic pathways were related to corneal graft rejection: amino acid biosynthesis and steroid hormone biosynthesis (Figure 2C). Elevated levels of the corresponding secondary metabolites – Hcy and 7α-hydroxypregnenolone, respectively – were found in the rejection group (Figure 2D).

### 3.2 Transcriptomics sequencing identified TRAF1, a key gene associated with Hcy-induced corneal graft rejection

**Figure 3:**
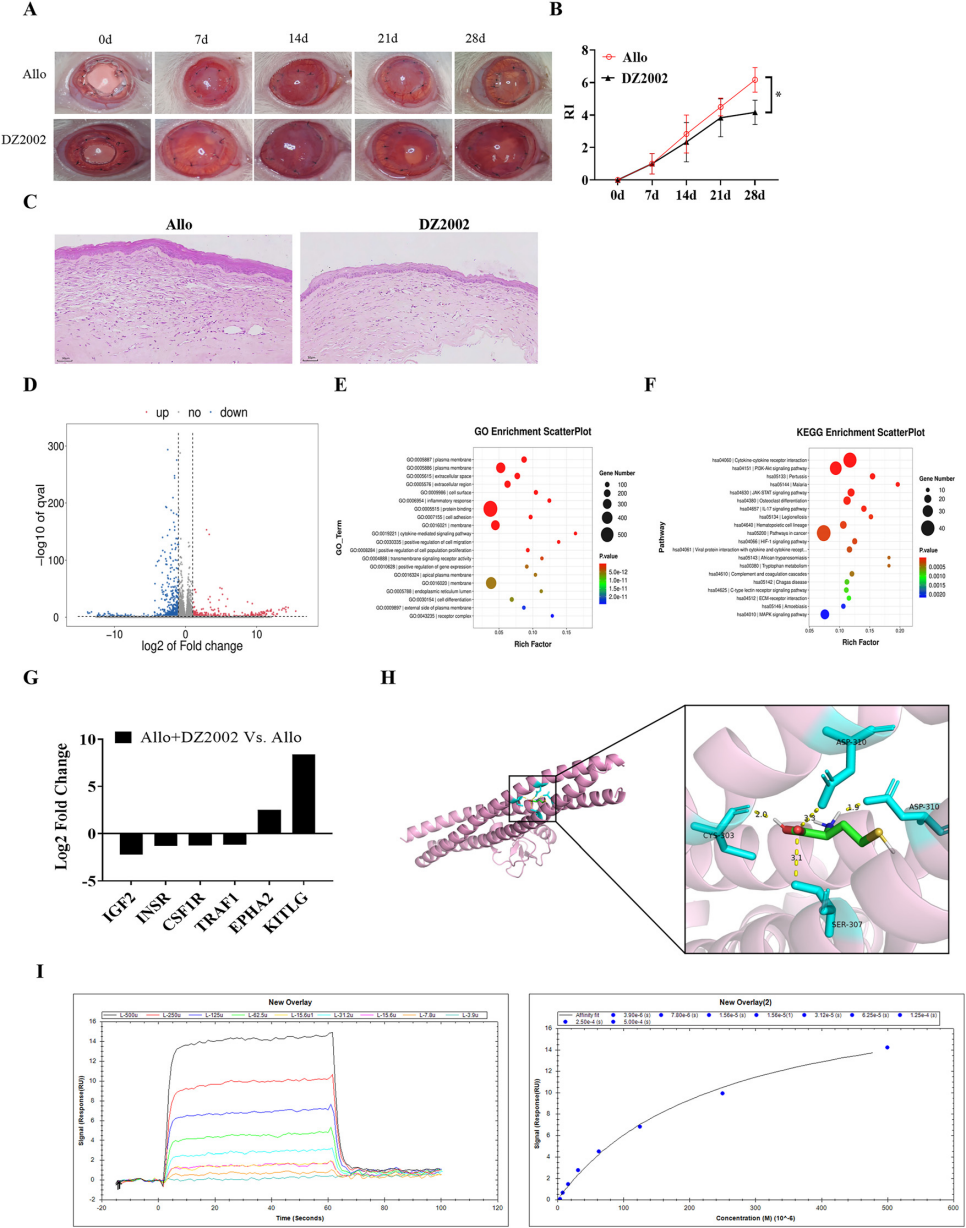
Screening of key genes associated with homocysteine intervention in corneal transplant rejection through transcriptomic sequencing. (A) Observation of corneal graft rejection under a stereomicroscope. (B) Statistical scoring of rejection severity. (C) H & E staining of corneal pathological changes (200 × magnification). (D) Volcano plot of differentially expressed genes. Analysis thresholds were set at |log_2_(Fold Change)| ≥ 1 and *p* value < 0.05. (E) GO enrichment analysis of differentially expressed genes. (F) KEGG enrichment analysis of differentially expressed genes. (G) Fold change distribution of differentially expressed genes. (H) Molecular docking model of TRAF1 with homocysteine. (I) SPR sensorgram (left) and SPR fitting curve (right).

Based on the results of metabolomics sequencing analysis, we used the Hcy inhibitor DZ2002 in the rejection model and found that DZ2002 significantly reduced the opacity and edema of the graft (Figure 3A). There was a significant reduction in the RI scores at 28 days post-surgery (Figure 3B), accompanied by a reduction in inflammatory cell infiltration as seen in the HE staining (Figure 3C).

To further analyze the mechanism of action, transcriptomics sequencing was performed on both the model group and the intervention group. A total of 967 differentially expressed genes were found in the model group, of which 293 were upregulated and 647 were downregulated (Figure 3D). GO and KEGG enrichment analyses were performed on these differentially expressed genes to identify the pathways related to immune rejection. Among the 292 pathways obtained, the PI3K/AKT pathway had the most significant difference (Figure 3E and F). Genes exhibiting a reverse change trend within these 48 pathways were scrutinized, leading to the identification of the following six key genes: IGF2, INSR, CSF1R, TRAF1, EPHA2, and KITLG (Figure 3G). After reviewing the literature, TRAF1 was finally selected for further investigation. Molecular docking revealed a binding site between Hcy and TRAF1 (Figure 3H).

To further confirm the interaction between TRAF1 and L-homocysteine, a surface plasmon resonance (SPR) assay was performed. The results are shown in the figure below. A series of L-homocysteine analyte concentrations (0 μM, 3.9 μM, 7.8 μM, 15.6 μM, 31.2 μM, 62.5 μM, 125 μM, 250 μM, and 500 μM) were applied to observe changes in the signal response. The sensorgram indicated that homocysteine stably binds to TRAF1. As the concentration decreased, the peak response also gradually decreased. The concentration–response fitting curve demonstrated that the signal response increased with rising analyte concentration, indicating enhanced binding affinity (Figure 3I), which suggests a dose-dependent binding relationship between homocysteine and TRAF1. The affinity constant (K_D_) calculated from the fitting curve was 2.46 × 10^−4^ M, confirming a measurable binding ability between homocysteine and TRAF1.

### 3.3 Hcy was found to activate the PI3K/AKT pathway through TRAF1 to promote Th17 differentiation and corneal graft rejection

#### 3.3.1 Identifying the possible inflammation-regulatory substances in the aqueous humor of rats treated with DZ2002 for corneal graft rejection

**Figure 4:**
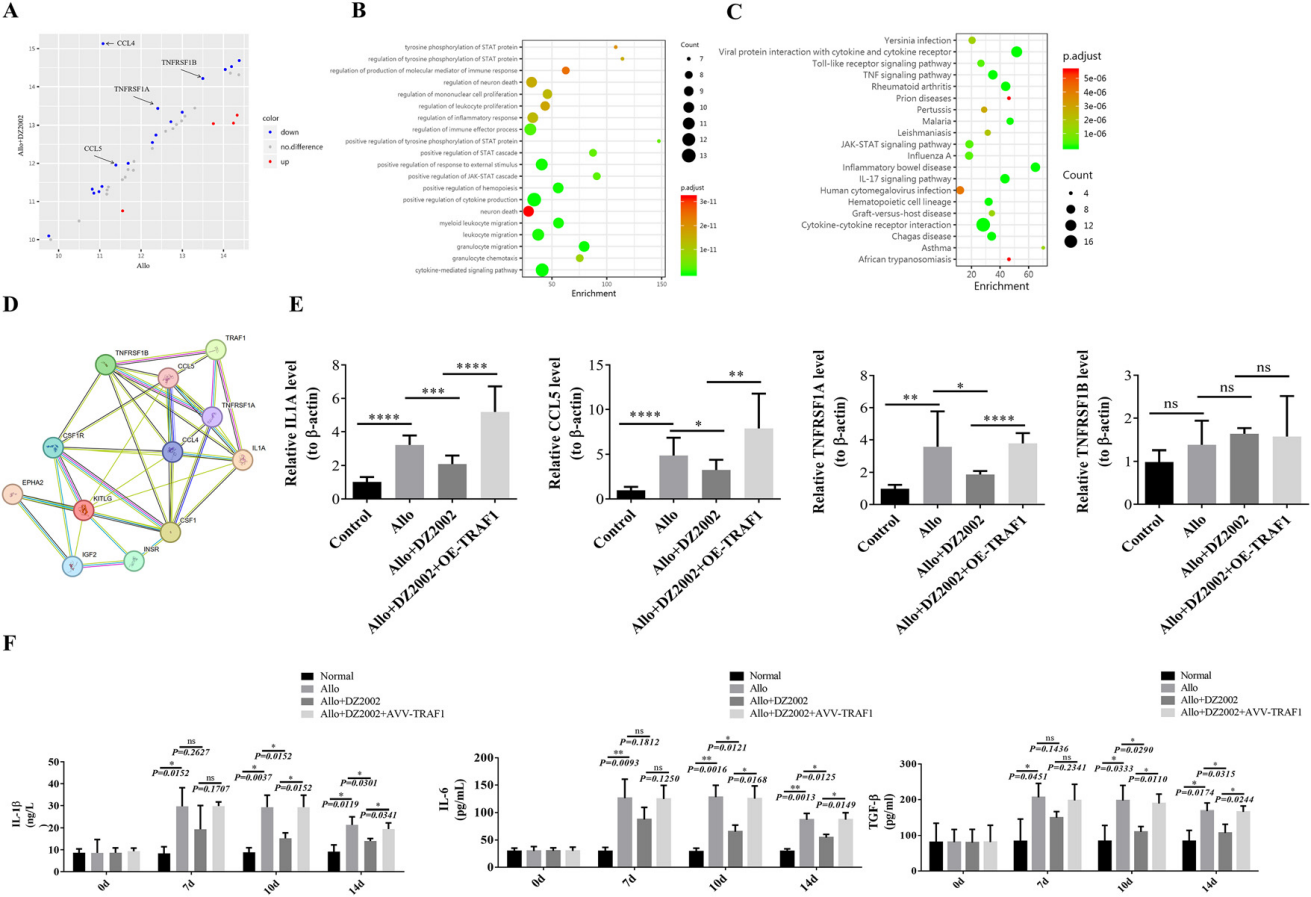
Screening of potential inflammatory mediators in aqueous humor following DZ2002 intervention in corneal rejection. (A) Expression profile of inflammatory factors detected by multiplex assay. (B) GO enrichment analysis of differentially expressed inflammatory factors. (C) KEGG enrichment analysis of differentially expressed inflammatory factors. (D) Protein-protein interaction (PPI) network between TRAF1 and relevant inflammatory factors. (E) RT-PCR validation of significantly altered inflammatory factors in corneal tissue, using *β*-actin as internal reference. (F) ELISA quantification of serum inflammatory factors IL-1β, IL-6, and TGF-β.

To clarify whether Hcy activates the PI3K/AKT pathway through TRAF1 to promote Th17 differentiation and corneal graft rejection, we first used a multiplex kit to detect the expression of inflammatory factors in the aqueous humor of rats treated with DZ2002 for corneal graft rejection and screened the following four factors related to TRAF1: IL1A, C–C motif chemokine ligand 5 (CCL5), TNFRSF1A, and TNFRSF1B (Figure 4A).

Subsequent GO and KEGG enrichment analyses of the differential inflammatory factors yielded a total of 20 significant signals, six of which were disease-related (Figure 4B and C). The Protein-Protein Interaction (PPI) network analysis demonstrated interactions between TRAF1 and these four factors (Figure 4D).

The expression levels of the four factors in corneal tissues were further detected using RT-PCR. This revealed higher expressions of IL1A, CCL5, and TNFRSF1A in the model group than in the control group. Conversely, the expressions of IL1A, CCL5, and TNFRSF1A were lower in the intervention group than in the model group. Additionally, the expressions of IL1A, CCL5, and TNFRSF1A in the intervention + OE-TRAF1 group were higher than those in the intervention group, whereas notably, TNFRSF1B expression did not differ significantly across groups (Figure 4E).

Furthermore, the levels of inflammatory factors IL-1β, IL-6, and TGF-β were detected using ELISA. There was a significant increase in the levels of IL-1β, IL-6, and TGF-β in the model group starting on day 7 post-surgery. Conversely, in the intervention group, the levels of IL-1β, IL-6, and TGF-β decreased after day 14, and in the intervention + OE-TRAF1 group, the levels of IL-1β, IL-6, and TGF-β increased again after day 14 (Figure 4F).

#### 3.3.2 Hcy activated the PI3K/AKT pathway through TRAF1 to promote corneal graft rejection

The effect of simultaneous intervention with DZ2002 and TRAF1 on corneal graft rejection was subsequently observed.

**Figure 5:**
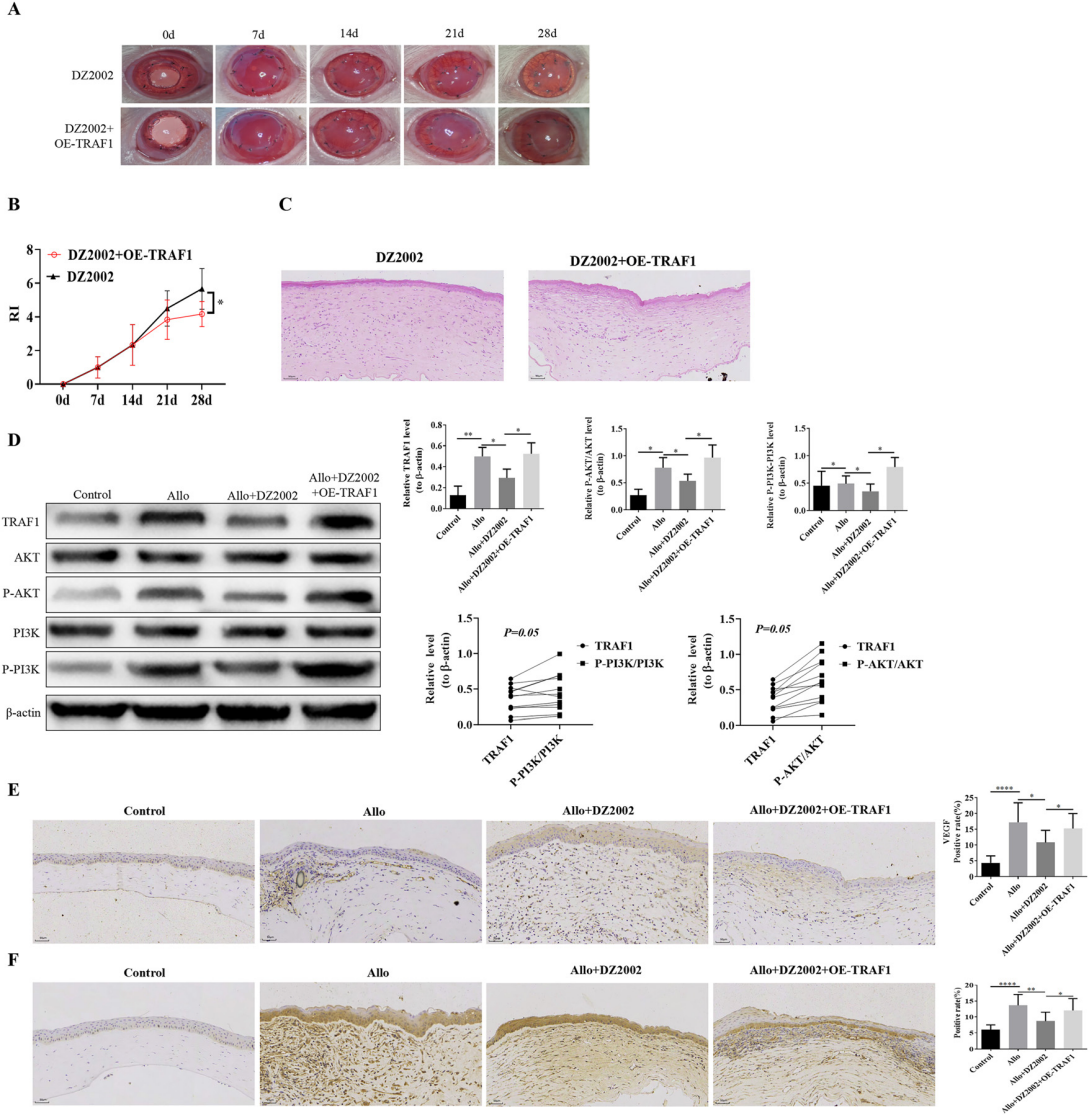
Effects of combined DZ2002 and TRAF1 intervention on corneal allograft rejection (A) Stereomicroscopic evaluation of corneal graft rejection. (B) Statistical scoring of rejection severity. (C) H & E staining demonstrating corneal pathological changes (200 × magnification). (D) Western blot analysis of TRAF1, PI3K, and AKT protein expression levels, with *β*-actin as the loading control. (E) Immunohistochemical (IHC) detection of VEGF positive rate (200 × magnification). (F) TUNEL staining for assessment of apoptotic levels (200 × magnification).

Compared with the DZ2002 group, the corneal graft in the DZ2002 + OE-TRAF1 group had increased opacity and edema, as observed under a stereomicroscope (Figure 5A). The RI scores were significantly elevated at four weeks (Figure 5B), and HE results showed that the inflammatory response was aggravated in the DZ2002 + OE-TRAF1 group (Figure 5C). WB analysis verified that the protein expressions of TRAF1, p-PI3K/PI3K, and p-AKT/AKT were significantly increased in the model group, whereas the protein expression levels of TRAF1, p-PI3K/PI3K, and p-AKT/AKT decreased after DZ2002 intervention. However, the expressions of TRAF1, p-PI3K/PI3K, and p-AKT/AKT were reversed following simultaneous intervention with DZ2002 and TRAF1 (Figure 5D). The effects of simultaneous intervention with DZ2002 and TRAF1 on angiogenesis and apoptosis levels were also additionally examined. Compared with the DZ2002 group, the VEGF expression levels and apoptosis rates were significantly higher in the DZ2002 + OE-TRAF1 group (Figure 5E and F). These results suggest that Hcy induced the activation of the PI3K/AKT pathway by promoting the expression of TRAF1, thereby leading to corneal graft rejection.

#### 3.3.3 In vitro cell experiments confirmed that Hcy activates the PI3K/AKT pathway through TRAF1 to promote Th17 differentiation

**Figure 6:**
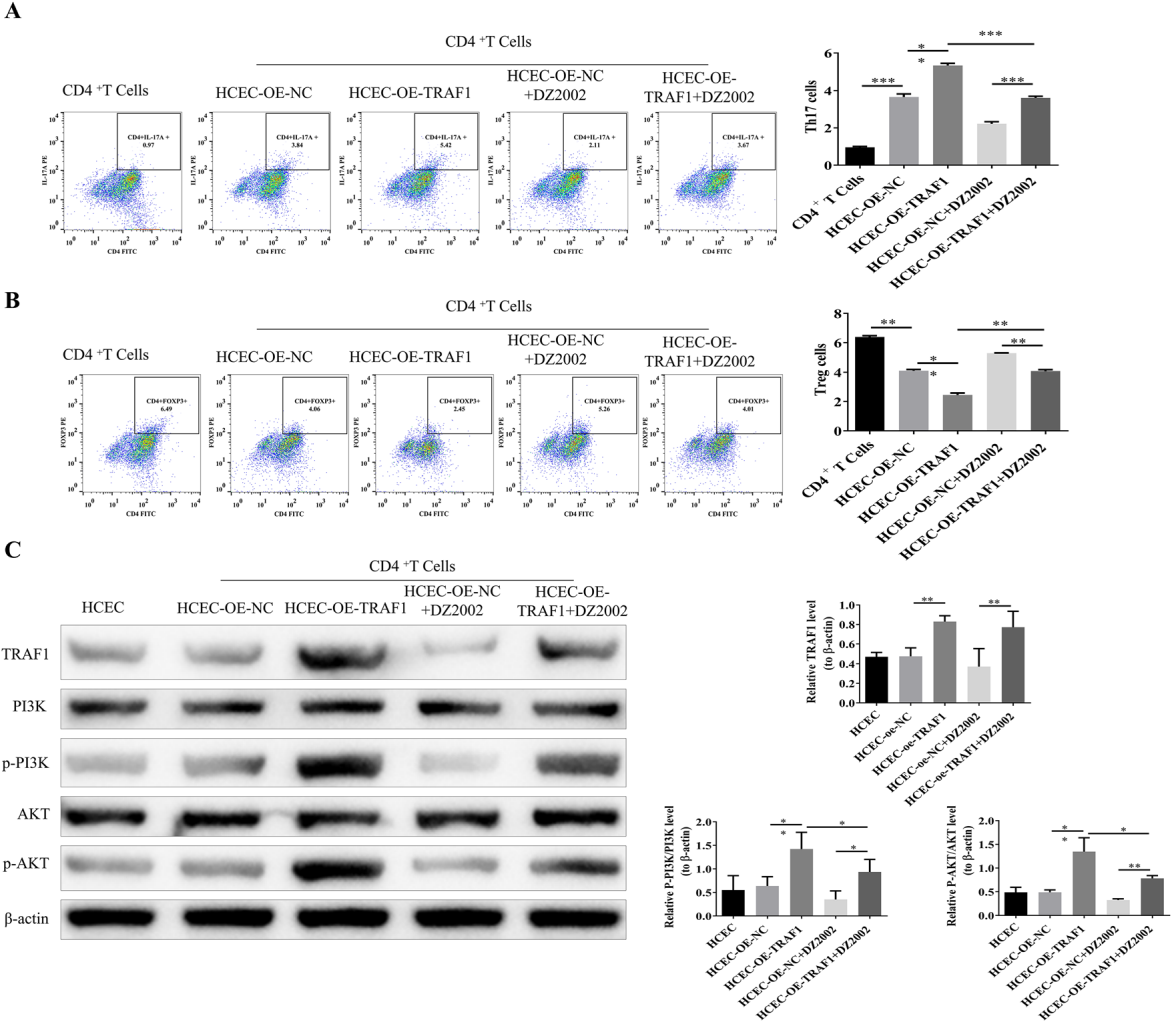
Mechanistic insights into Hcy-induced corneal rejection via TRAF1-mediated activation of the PI3K/AKT pathway (A) Flow cytometric analysis of TH17 cell proportion. (B) Flow cytometric analysis of Treg cell proportion. (C) Western blot analysis of TRAF1, PI3K, and AKT protein expression, using *β*-actin as the loading control.

To explore the mechanism of corneal graft rejection, *in vitro* cell experiments were conducted to validate that Hcy activates the PI3K/AKT pathway through TRAF1. Given the pivotal role of CD4+ T cell polarization in immune imbalance, the effect of human corneal epithelial cells (HCEC) on CD4+ T cell polarization post-DZ2002 and TRAF1 intervention was examined via flow cytometry. The results showed that, in the HCEC + OE-TRAF1 group compared with the HCEC + OE-NC group, Th17 cells increased whereas Treg decreased. Also, compared with the HCEC + OE-NC + DZ2002 group, Th17 cells increased and Treg decreased in the HCEC + OE-TRAF1 + DZ2002 group, indicating that the increased expression level of TRAF1 could promote the differentiation of CD4+ T cells into Th17. In the HCEC + OE-TRAF1 + DZ2002 group, compared with the HCEC + OE-TRAF1 group, Th17 cells decreased and Treg increased, suggesting that DZ2002 could inhibit the regulation of CD4 cell polarization by TRAF1 (Figure 6A and B).

WB was used to assess the protein expression levels of PI3K and AKT in HCEC cells co-cultured with CD4+ T cells. The results showed that, compared with the HCEC + OE-NC group, the expressions of TRAF1, p-PI3K/PI3K, and p-AKT/AKT were significantly increased in the HCEC + OE-TRAF1 group. Decreased levels of TRAF1, p-PI3K/PI3K, and p-AKT/AKT were observed in the HCEC + OE-TRAF1 + DZ2002 group when compared with the HCEC + OE-NC + DZ2002 group (Figure 6C). These results suggest that Hcy activates the PI3K/AKT pathway by upregulating TRAF1, thereby contributing to corneal graft rejection and increased Th17 differentiation.

## 4 Discussion

The treatment of corneal graft rejection aims to restore the normal metabolic processes of corneal tissue and preserve corneal graft transparency. A primary focus of research in this area revolves around reducing the destructive attack of CD4+ T cells on the graft [8], [9], [10]. Strategies such as injecting the *alpha-melanocyte stimulating hormone* (α-MSH), vasoactive intestinal peptide, transforming growth factor β2 (TGF-β2), and thromboxane-1 (TSP-1) into the anterior chamber have been explored to inhibit lymphatic production and regulate T cell activity through different signaling pathways, thereby maintaining the immune privilege of the anterior chamber [11]. Although these approaches have shown promising results, they are still associated with challenges such as high cost, technical complexity, and limited scalability.

When a corneal graft rejection occurs, the patient’s immune system identifies the transplanted corneal tissue as a foreign substance and initiates inflammatory responses to eliminate it. Th1, Th2, and Th17 (CD4+ CD25+ RORγt+) cells play crucial roles in this inflammatory cascade, releasing a variety of cytokines and chemical factors such as tumor necrosis factor-α (TNF-α), interleukin-1β (IL-1β), interleukin-2 (IL-2), interleukin-6 (IL-6), interleukin-17 (IL-17), TGF-β, and interleukin-21 (IL-21). These factors further regulate the immune response and inflammation, accompanied by massive corneal neovascularization and increased VEGF factor expression [12], [13], [14], [15]. Consistent with previous studies, in our study, HE staining of the corneal tissue with graft rejection showed significant infiltration of inflammatory cells. Also, levels of IL-1β, IL-6, and TGF-β were significantly elevated since day 7 post-surgery, while VEGF expression and corneal apoptosis were significantly increased at four weeks post-surgery.

The use of metabolomics in the investigation of corneal diseases represents a novel approach that offers new perspectives and methodologies for uncovering the pathogenesis, diagnosis, and treatment of these conditions. Corneal epithelial cells, stromal cells, and endothelial cells collectively engage in protein synthesis and metabolic processes crucial for maintaining the normal physiological functioning of the cornea. Any pathological state that interferes with corneal protein metabolism may lead to corneal opacity and vision loss [16].

### 4.1 The crucial role of Hcy in corneal graft rejection

In this study, metabolomics analysis performed on the serum of rats with rejection revealed two metabolic pathways related to corneal graft rejection: amino acid and steroid hormone biosynthesis. The corresponding secondary metabolites were Hcy and 7α-hydroxypregnenolone, respectively. However, no direct association has been reported between 7α-OH-DHEA and corneal diseases. Hcy is a sulfur-containing amino acid that is involved in vivo protein metabolism processes. It can be metabolized to methionine or reconverted to methionine through the action of vitamins B6, B12, and folic acid.

Abnormally elevated Hcy levels are associated with increased risks of a variety of diseases, including cardiovascular, neurological, and ocular diseases [17]. Although studies have linked Hcy to conditions such as keratoconus, dry eye, and corneal ulcers, there are relatively few studies in the field of corneal transplantation. However, there is emerging evidence suggesting that Hcy may influence immune cell function through multiple mechanisms, such as modulating cellular oxidative stress levels, and consequently indirectly affecting the activation and differentiation of T cells [18]. Notably, Wolos et al. [19] demonstrated that S-adenosylhomocysteine hydrolase inhibitors can affect immune rejection *in vitro* and *in vivo*. The results of this study underscore the role of Hcy as a key metabolite potentially implicated in promoting the occurrence of rejection through some mechanism, thus providing new clues for further exploration of the molecular mechanisms involved in corneal graft rejection.

### 4.2 Activation of TRAF1 and PI3K/AKT pathways

Post-corneal transplantation, rats was administered DZ2002 via eye drops at a frequency of four times per day. After four weeks of continuous use, transcriptomics sequencing was used to identify the key genes associated with Hcy influencing corneal graft rejection and found that the expression of TRAF1 was significantly reduced after the DZ2002 intervention. GO and KEGG enrichment analyses of the differentially expressed genes identified the PI3K/AKT pathway to be the most significant among the pathways related to immune rejection. Molecular docking analysis showed that there was a binding site between Hcy and TRAF1. We have successfully validated, for the first time, the specific binding between Hcy and TRAF1 protein using surface plasmon resonance (SPR). Therefore, we further speculated that DZ2002 inhibited the binding of Hcy to TRAF1 during corneal graft rejection.

The exact mechanism of TRAF1 in CD4+ T cell differentiation and function is still being studied. It is thought to be an important molecule in regulating CD4+ T cell-mediated immune responses and possible immunopathological processes [20], 21]. A variety of intracellular signaling pathways regulate Th17 cell differentiation, including the complex transcription factor network involving PI3K, AKT, and mTOR [22], [23], [24]. However, the increased expression of these inflammatory indicators after the addition of the TRAF1AVV virus in the DZ2002 group indicates that DZ2002 mainly reduced the inflammatory response by downregulating the expression of TRAF1.

Immune cells, notably CD4+ T cells, migrate to corneal grafts – a process that constitutes a fundamental step in the cascade leading to immune rejection. Chemokines not only attract the migration and localization of CD4+ T cells but also affect their differentiation and exert partial immune regulatory effects, thus playing a key role in the immune response after corneal transplantation [25]. In the current study, we employed a multiplex kit to detect the expression levels of inflammatory factors in the aqueous humor of rats treated with DZ2002 for graft rejection, and this identified four inflammatory cytokines, namely, IL1A, CCL5, TNFRSF1A, and TNFRSF1B, associated with TRAF1. These findings offer important clues for further research on the role of Hcy in corneal graft rejection. In this study, GO and KEGG enrichment analyses of differential inflammatory factors were performed, and RT-PCR results verified that IL1A, CCL5, and TNFRSF1A were significantly reduced in the DZ2002 group. Meanwhile, ELISA revealed a reduction in all the inflammatory factors, i.e., IL-1β, IL-6, and TGF-β, underscoring the significant impact of DZ2002 on inhibiting the inflammatory response of corneal transplantation.

The inflammatory regulatory role of TRAF1 is cell type- and pathology-dependent. It primarily exerts anti-inflammatory effects in cardiomyocytes, macrophages, and T cells, while exhibiting pro-inflammatory effects in endothelial cells and certain tumor models. The underlying mechanisms involve NF-κB and MAPK signaling pathways, as well as interactions with cIAP2 [26], 27]. This dual functionality may be attributed to differences in the adapter molecules that TRAF1 interacts with across distinct cell types [28].

In this study, overexpression of TRAF1 on the basis of DZ2002 intervention significantly upregulated the expression of IL-1A, CCL5, and TNFRSF1A in corneal tissue. Furthermore, levels of the inflammatory factors IL-1β and IL-6, as well as TGF-β, were markedly elevated in aqueous humor. TGF-β is a multifunctional cytokine produced and secreted by a wide range of cell types, demonstrating broad cellular origins and functional diversity. It is primarily secreted by M2 macrophages, dendritic cells (DCs), T cells, as well as various epithelial and vascular cells [29], [30], [31]. The results indicate that TRAF1 promotes the expression of inflammatory factors in corneal rejection and activates the secretory functions of both immune cells and corneal epithelial cells.

Studies have shown that the damaged corneal tissues with inflammation after corneal transplantation would express a series of chemokines, including CCL2, CCL3, CCL4, CCL5, and CXCL10 [25], 32], 33]. In this study, our focus was on the examination and validation of CCL5, a TRAF1-related chemokine. Our findings suggest that TRAF1 has a potential role in promoting rejection by increasing the expression of CCL5. CCL5, also known as Regulated upon Activation, Normal T Cell Expressed and Secreted (RANTES), is a small pro-inflammatory cytokine belonging to the CC chemokine family. It is secreted by various cells, including T cells, macrophages, and fibroblasts, and plays an important role in immune responses and inflammatory processes [34]. Mechanistically, CCL5 exerts its effects by binding to specific chemokine receptors such as CCR1, CCR3, and CCR5. It has a strong chemotaxis effect on various immune cells, including monocytes, T cells, eosinophils, and so on, thereby facilitating their migration to sites of inflammation or infection.

In the present experiment, it was observed that the phosphorylation level of PI3K/AKT and CCL5 was decreased in the DZ2002 group, suggesting that the activation of the PI3K/AKT pathway was inhibited. Consequently, VEGF expression and corneal apoptosis rates were reduced. Additionally, in the co-culture of corneal epithelial cells and CD4+ T cells, we confirmed that DZ2002 could inhibit the differentiation of CD4+ T cells into Th17 by downregulating the expression of TRAF1.

### 4.3 Potential therapeutic effects of DZ2002

The effects of Hcy on immune regulation may be attributed to its involvement in several of the following underlying mechanisms: Elevated Hcy levels can increase oxidative stress in the body, activating immune cells such as macrophages and lymphocytes. This may lead to an exacerbation of the inflammatory response, thereby resulting in cell damage. This oxidative stress can increase the production of inflammatory mediators, including cytokines and chemokines [35], 36], which regulate immune cell activity [37], 38] and indirectly affect the immune response by influencing vascular endothelial cell function [39], [40], [41], [42]. Additionally, Hcy has been implicated in the development of autoimmune diseases [43], 44]. The *in vivo* metabolism of Hcy involves methylation processes and aberrations in these may affect gene expression patterns, including those that regulate the immune system, thereby affecting the function and development of immune cells [45].

DZ2002 has been shown to modulate the STAT3-PI3K-Akt-NF-κB signaling pathway, resulting in reduced expression of genes associated with angiogenesis and decreased levels of inflammation in the cornea and conjunctiva. This modulation helps alleviate dry eye symptoms [46]. Furthermore, in this present study, DZ2002 effectively inhibited corneal graft rejection, suggesting that DZ2002 may be a potential drug for the treatment of corneal graft rejection.

Although some progress has been made in this study, there are still many issues that warrant further exploration. For example, uncovering the specific mechanisms of action of the Hcy, TRAF1, and PI3K/AKT pathways in corneal graft rejection requires a more in-depth study. Additionally, its application potential and safety profile in clinical practice need further investigations.

One limitation of this study should be noted. The absence of a syngeneic transplantation control group (e.g., SD rats to SD rats) makes it difficult to entirely rule out the potential influence of non-specific surgical trauma-induced inflammation on the early observed outcomes. Nevertheless, the progressively worsening pathological process, the specific molecular profile highly consistent with allogeneic immune responses, and the significant therapeutic effect of DZ2002 collectively indicate that the core findings of this study are primarily attributable to allogeneic immune rejection rather than merely postoperative inflammation [47]. Future studies should incorporate a syngeneic control group to establish a definitive baseline and provide critical evidence for further validating the specific role of the Hcy–TRAF1 axis in rejection responses.

## 5 Conclusion

In this study, employing a metabolomic approach, the metabolite Hcy and the differentially expressed gene TRAF1 were identified in the context of their role in corneal graft rejection. Experiments using a rat model of graft rejection confirmed that inhibiting Hcy led to a decrease in TRAF1 expression, lower levels of the chemokine CCL5 in the aqueous humor, suppression of the differentiation of T cells into Th17, and a subsequent reduction in the rejection index. A preliminary exploration of the DZ2002-related mechanisms involved in alleviating rejection revealed that homotype DZ2002 was effective in reducing rejection, offering promise for this to emerge as a new drug for the treatment of corneal graft rejection.
